# Treatises on Physiology

**Published:** 1887-12

**Authors:** 


### BOOK REVIEWS.

TREATISES ON PHYSIOLOGY.

*For the use of students and practitoners of medicine.* By Henry C. Chapman, M. D., Professor of Institutes of Medicine and Medical Jurisprudence in Jefferson Medical College, of Philadelphia. Pp. xv. and 945. Philadelphia: Lea Brothers & Co. 1887.

The feeling that a new work on such a well-worn subject as physiology must introduce itself with an apology for its existence, was fully appreciated by the author of this one. The excuse given in the preface is, that while the many other text-books in this branch of medical science are excellent, experience gained as a teacher of the subject, convinced him that one was needed, "based upon comparative and pathological anatomy, clinical medicine, physics and chemistry, as well as upon experimental research."

In all cases, which admit of it, what is known as the historical method or order is employed in this book—a plan which deserves commendation. The author seems to have known clearly what he desired to do, and to have done it well. Very free use has been made of illustrations taken from other books, but in each case acknowledgement is made, and the new cuts are sufficiently numerous to make the illustrations, as a whole, very satisfactory. The author's style is so involved that many students will find it difficult to understand some of his descriptions, whilst others are clear enough.

He apparently ignores, or does not credit the results of some recent observations upon the function of the saliva, and upon the superior costal respiration in the female. E. W.

